# Anti-Spike and Neutralizing Antibodies after Two Doses of COVID-19 Sinopharm/BIBP Vaccine

**DOI:** 10.3390/vaccines10081340

**Published:** 2022-08-18

**Authors:** Eman A. Omran, Roaa E. El Naggar, Logina A. Ezz Elarab, Mona H. Hashish, Mohammed A. El-Barrawy, Ibrahim A. Abdelwahab, Marwa M. Fekry

**Affiliations:** 1 Department of Microbiology, High Institute of Public Health, Alexandria University, 165 El-Horreya Avenue, El-Ibrahimia, Alexandria 21524, Egypt; monash64@alexu.edu.eg (M.H.H.); dr.elbarrawy@alexu.edu.eg (M.A.E.-B.); hiph.mfekry@alexu.edu.eg (M.M.F.); 2 Ministry of Health and Population, Cairo 11516, Egypt; hiph.elnaggar@alexu.edu.eg (R.E.E.N.); hiph.ezzelarab@alexu.edu.eg (L.A.E.E.); 3 Microbiology and Immunology Department, Faculty of Pharmacy, Pharos University in Alexandria, Alexandria 21311, Egypt; ibrahim.abdelwahab@pua.edu.eg

**Keywords:** humoral immunity, neutralizing antibodies, COVID-19 vaccines, SARS-CoV-2

## Abstract

Host response to COVID-19 vaccines is partially evaluated through the estimation of antibody response, specifically the binding anti-spike (anti-S) and the neutralizing antibodies (nAbs) against SARS-CoV-2. Vaccine-induced humoral response affects decisions on the choice of vaccine type, vaccine acceptance, and the need for boosting. Identification of risk factors for poor antibody response helps to stratify individuals who might potentially require booster doses. The primary objective of this cross-sectional study was to investigate the antibody response after receiving two Sinopharm vaccine doses. Factors affecting antibody response were additionally studied. Moreover, a predictive cutoff for anti-S was generated to predict positivity of nAbs. Blood samples were collected from 92 adults and relevant data were recorded. Antibody levels (anti-S and nAbs) against SARS-CoV-2 were tested one month following the second dose of Sinopharm vaccine using two commercial ELISA tests. Among the 92 participants, 88 tested positive for anti-S (95.7%), with a median level of 52.15 RU/mL (equivalent to 166.88 BAU/mL). Fewer participants (67.4%) were positive for nAbs, with a median percentage of inhibition (%IH) of 50.62% (24.05–84.36). A significant positive correlation existed between the titers of both antibodies (correlation coefficient = 0.875, *p* < 0.001). When the anti-S titer was greater than 40 RU/mL (128 BAU/mL), nAbs were also positive with a sensitivity of 80.6% and a specificity of 90%. Positive nAbs results were associated with a higher anti-S titers (62.1 RU/mL) compared to negative nAbs (mean anti-S titer of 18.6 RU/mL). History of COVID-19 infection was significantly associated with higher titers of anti-S (*p* = 0.043) and higher IH% of nAbs (*p* = 0.048). Hypertensive participants were found to have significantly higher median titers of anti-S (101.18 RU/mL) compared with non-hypertensive ones (42.15 RU/mL), *p* = 0.034. Post-vaccination headache was significantly higher among those with higher anti-S than those with relatively lower titers (98.82 versus 43.69 RU/mL, *p* = 0.048). It can be concluded that the Sinopharm vaccine produced high levels of binding antibodies but with low neutralizing abilities. Also, levels of anti-S titer greater than 40 RU/mL could adequately predict positivity of nAbs without need for their testing.

## 1. Introduction

The coronavirus disease 2019 (COVID-19) pandemic had tremendous public health impacts. As of 29 May 2022, over 526 million COVID-19 confirmed cases and over six million deaths have been reported to the World Health Organization (WHO) globally. Mathematical modeling plays an important role to better understand the disease dynamics and designing strategies to manage quickly spreading infectious diseases [1,2]. The global impact of the COVID-19 pandemic has resulted in an unprecedented level of public interest in vaccines as a major pillar of reducing infections and mortalities. As of 29 May 2022, a total of 11,811,627,599 vaccine doses have been administered worldwide [3]. However, reports of adverse events have led some people to express concerns about getting vaccinated, delay getting vaccinated, or be strongly opposed to vaccination [4].

In Egypt, anti-COVID vaccines were first available in January 2021 and were solely administered to individuals working in the healthcare sector, particularly those working in hospitals for COVID-19 isolation and pulmonology hospitals. Vaccination of the general population started in April 2021 and the first two available vaccines in Egypt were Sinopharm and Oxford–AstraZeneca [5]. The BIBP-CorV (Sinopharm’s Beijing Institute of Biological Products, Beijing, China) vaccine is an inactivated whole virus vaccine produced in Vero cells, with an aluminum hydroxide adjuvant [6]. A large Phase III trial has shown that two doses, administered at an interval of 21 days, had an efficacy of 79% against symptomatic SARS-CoV-2 infection and against hospitalization [7]. Sinopharm was given an emergency user license by the WHO on 7 May 2021 and was included in the Global Alliance for Vaccines and Immunizations (GAVI), to be distributed under the COVAX program [8].

The spike (S) protein on the surface of SARS-CoV-2 virion mediates receptor recognition and membrane fusion with human angiotensin-converting enzyme 2 (ACE2) molecules. Antibodies against the S antigen (anti-S) have a protective role, as they prevent viral binding and entry. Subsets of anti-S immunoglobulins have a neutralizing ability [9]. According to the WHO, within 4 weeks following infection, 90–99% of individuals infected with the SARS-CoV-2 virus develop detectable neutralizing antibodies (nAbs) [10]. Virus-specific nAbs, as a correlate of protection for symptomatic COVID-19 infection, are an important standard to evaluate the efficacy of vaccines [11,12]. Following vaccination, high titers confer stronger and more durable immunity compared to lower antibody titers. However, a consensual cutoff titer of nAbs as a correlate of protection has not been defined yet [13].

Identification of vaccine-induced immune response affects the choice of vaccine types being supplied by different countries. It also is an important determinant of vaccine acceptance in the community. Host factors affecting the vaccine-induced antibody response should be evaluated in order to identify high-risk groups with low antibody levels who are a priority for booster dose provision. Owing to the technically easier testing of anti-S compared to the testing of nAbs, identification of cutoff level for anti-S that predicts nAbs seropositivity might be more meaningful in terms of seroprotection.

Our study aimed at evaluating the levels of antibody production (anti-spike and nAbs) after a double dose of Sinopharm vaccination and identifying possible factors associated with immune response including vaccine side effects. Evaluation of the correlation between both antibodies was performed and a cutoff of anti-S was generated that could adequately predict nAb positivity.

## 2. Materials and Methods

### 2.1. Study Setting

This cross-sectional study was carried out from January 2021 through June 2021, which coincided with the end of the second until the end of the third wave of COVID-19 in Egypt. At the time of our study, vaccines were reserved primarily for healthcare workers (HCWs). Booster doses were not provided as per the national vaccination protocol since priority was given to wider coverage of double vaccine dosages. The national vaccination coverage (double doses) at the time of our sample collection was 0.7% [14].

### 2.2. Sample Size

Based on a study on Sinopharm COVID-19 vaccine [6], a minimal total sample size of 78 subjects was needed to detect positive SARS-CoV-2 antibodies after receiving the second dose of vaccine, using One Proportion Power Analysis in NCSS & PASS Program that achieves 80% power with a target significance level at 5%.

### 2.3. Data Collection Methods and Tools

Our study was conducted on 92 Egyptian adults, 18–60 years old, one month after receiving the second dose of the Sinopharm COVID-19 vaccine. Participants were recruited through web-based invitations on social media platforms. Participants with the following criteria were excluded: those receiving chemo- or radiotherapy, immunosuppressive agents, or systemic corticosteroids. The study was approved by the Ethics Committee of the High Institute of Public Health (HIPH). Written consent was taken from each vaccine recipient after explaining the purpose of the study.

A predesigned structured questionnaire was completed for each participant, which included sociodemographic data, medical history of comorbidities, and COVID-19 infection, as well as the occurrence of any adverse reactions within one week following vaccination. Using aseptic techniques, blood samples of 3 mL were collected from each participant for antibody detection. Samples were centrifuged at 3000 rpm, and serum was stored at −20 °C until testing for SARS-CoV-2 antibodies. The anti-SARS-CoV-2 Quantivac enzyme-linked immunosorbent assay (ELISA) (EUROIMMUN, Lübeck, Germany) was used to detect immunoglobulin class IgG against the S1 domain of the viral spike protein of SARS-CoV-2, while nAbs were tested using the semi-quantitative SARS-CoV-2 NeutraLISA kit (EUROIMMUN, Lübeck, Germany). Tests were performed according to the manufacturer’s instructions.

According to the manufacturer, negative results were those with titers <8 RU/mL, borderline results ranged between 8 to <11 RU/mL, while positive results were those with titers ≥11 RU/mL. In our study, borderline results were added to the negative results for a more meaningful statistical analysis.

For our study, some of our anti-S results (RU/mL) were additionally expressed as Binding Antibody Unit/mL (BAU/mL) by multiplying the results in RU/mL by a conversion factor of 3.2 (as stated by the manufacturer). This was done in accordance with the recommendations of the WHO first International Standard (IS) for anti-SARS-CoV-2 immunoglobulin (NIBSC code 20/136) for ensuring comparability between different antibody test kits for SARS-CoV-2 [15,16]. This allowed for the comparison of our results with other studies that used other types of kits to test for anti-S.

The neutralizing antibody (nAbs) test (NeutraLISA, Euroimmun, Lübeck, Germany) is a surrogate virus neutralization test to detect immunoglobulins (Igs A, M, and G) that can neutralize SARS-CoV-2 via inhibition of S1/RBD binding to ACE2 receptors [17]. According to the manufacturer, the specificity of this test is 99.7% and its sensitivity is 95.9%. According to the manufacturer’s instructions, results of percentage inhibition (%IH) < 20 were considered “negative”, %IH > 20 to <35 were “borderline”, while %IH > 35 indicated a “positive” result [17]. For easier statistical analysis, borderline results were added to the negative results.

### 2.4. Statistical Analysis

Statistical analysis was carried out using SPSS statistics software version 24 (SPSS, Inc., Chicago, IL, USA). Quantitative data were tested for normality using the Kolmogorov–Smirnov test. The “age” variable was not normally distributed; it was described in terms of median and range. Non-parametric statistical tests of significance were applied; the Kruskal–Wallis test was used to compare three independent groups. Qualitative data were expressed by numbers and percentages. Pearson’s chi-squared test was used to test the association between qualitative variables. The receiver operating characteristic (ROC) curve analysis was submitted to Med-Calc program to test the sensitivity, specificity, and cutoff values, which are the fundamental tools for diagnostic test evaluation. In all other applied statistical tests of significance, a *p*-value (<0.05) was considered significant.

## 3. Results

The median (interquartile range; IQR) age of the participants was 39 (33–45) years. Males were predominant (58.7%), while females comprised 41.3% of participants. The majority of participants were physicians (46.7%), followed by technicians (20.7%) and workers (12.0%). Smoking was prevalent in 21.7% (n = 20) of participants, while 10.9% were hypertensive and 6.5% were asthmatic. Equal rates (4.3%) were recorded for the prevalence of diabetes mellitus, thyroid disorders, and musculoskeletal diseases. Most participants (82.6%) did not report a previous COVID-19 infection, while only 17.4% reported a history of COVID-19 infection before being vaccinated. Most of the previously infected participants (43.8%) were diagnosed based solely on their symptoms, while 31.2% had additional laboratory investigations done, and only 25% additionally received a chest CT. Most participants (96.7%) were vaccinated to protect themselves from COVID-19 infection, while only 3.3% were vaccinated as mandated by their workplaces.

As regards vaccine side-effects, 35.9% of them recorded one or more of the following symptoms: fever (n = 6, 6.5%), fatigue (n = 10, 10.9%), headache (n = 12, 13.0%), pain at the site of injection (n = 16, 17.4%), arthralgia/myalgia (n = 2), and nausea (n = 1).

**Figure 1:**
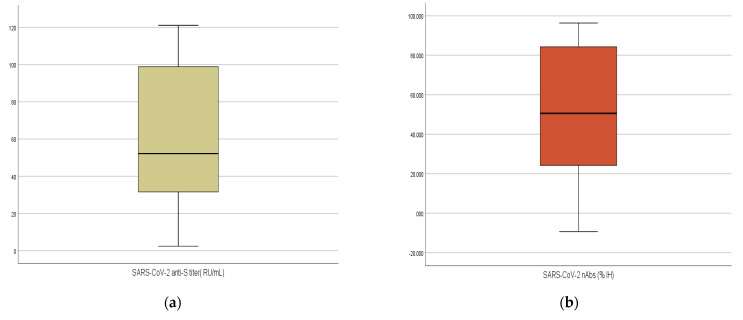
(**a**,**b**): Boxplot illustrating the differences in the titers of SARS-CoV-2 anti-S and %IH of nAbs of 92 Sinopharm vaccine recipients.

Among the 92 participants, 88 tested positive for anti-S (95.7%). The overall median (IQR) titer of anti-S titer was 52.15 (31.44–99.12) RU/mL, with a range of 2–120 RU/mL. This titer was equivalent to 166.88 BAU/mL. Most participants (67.4%) were positive for nAbs. The median (IQR) of neutralization inhibition (IH) % was 50.62% (24.05–84.36), and it ranged from 9.24 to 96.34 (Table 1 and Figure 1).

Of the 92 vaccinated participants, 62 (67.4%) tested positive for both anti-S and nAbs. Among those with positive anti-S results, 26 participants (n = 29.5%) were negative for nAbs. None of those with positive nAbs had a negative result for anti-S (Table 1).

**Figure 2:**
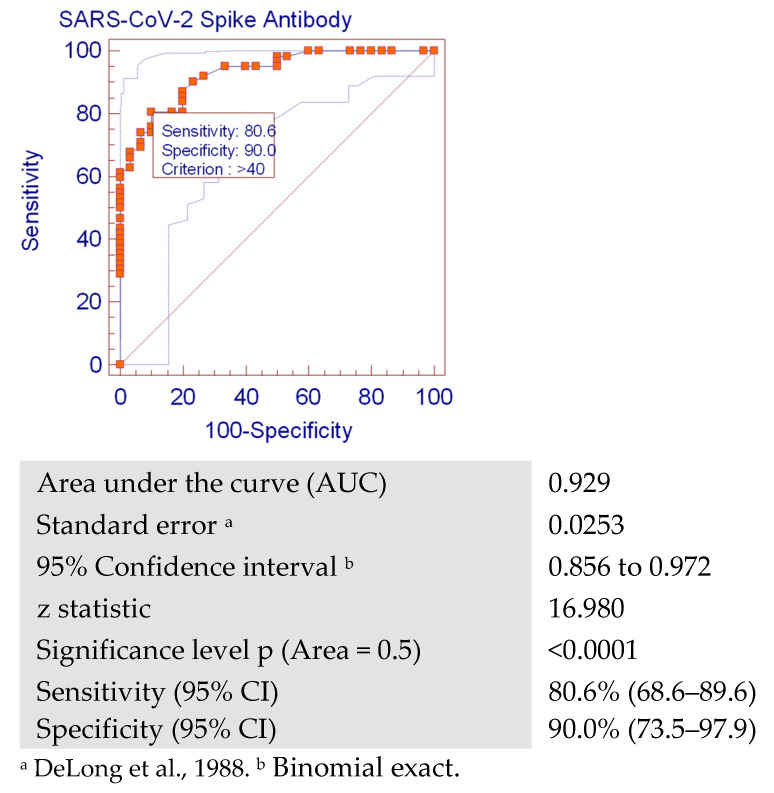
Receiver operating characteristic (ROC) curve analysis showing the cutoff value for SARS-CoV-2 anti-S predictive for positivity of neutralizing antibodies (with a sensitivity of 80.6% (68.6–89.6) and specificity of 90.0% (73.5–97.9)).

As shown in the ROC curve, anti-S was found to be a good indicator for the cases with positive nAbs (area under the curve = 0.929). The ROC showed that when the anti-S titer was greater than 40 RU/mL (128 BAU/mL), nAbs were also positive, with sensitivity of 80.6% (95% CI: 68.6%–89.6%) and specificity of 90% (95% CI: 73.5%–97.9%) (Figure 2).

Only 70.5% of anti-S-positive participants were also positive for nAbs, with a significant difference (*p* = 0.003) between the results of the anti-S and nAbs results (Table 2). Participants with positive results for both antibodies had a mean positive anti-S titer of 62.1 RU/mL, while a lower mean titer of anti-S (18.6 RU/mL) was recorded among participants with positive anti-S and negative nAbs.

A significant positive correlation was seen between the titers of both antibodies (correlation coefficient = 0.875, *p* < 0.001) (Table 3).

Age was not significantly correlated with the levels of any of the antibodies (with a negative correlation seen between age and levels of antibodies). History of COVID-19 infection was significantly associated with higher titers of anti-S (*p* = 0.043) and higher IH% of nAbs (*p* = 0.048) (Table 4). Gender and smoking were neither associated with seropositivity (Table 5) nor with the levels of either of the antibodies (Table 4).

Hypertensive participants were found to have significantly higher median titers of anti-S (101.18 RU/mL) compared with non-hypertensive ones (42.15 RU/mL), *p* = 0.034. Other comorbidities (diabetes, thyroid diseases, lung conditions, and allergy) were not associated with significant changes in anti-S or nAbs (Table 6 and Table 7).

None of the post-vaccination side effects was significantly associated with seropositivity of either SARS COV-2 anti-S or nAbs (Table 8). Headache was significantly higher among those with higher anti-S than those with relatively lower titers (98.82 versus 43.69 RU/mL, *p* = 0.048) (Table 9).

## 4. Discussion

Despite the globally declining pandemic [3,18], evaluating the efficacy of different COVID-19 vaccines is still required for fear of the evolution of new viral variants and the rising of COVID-19 cases. According to the WHO (2022), appropriately designed immuno-bridging studies are an acceptable alternative approach for authorizing vaccines. Neutralizing antibody titers may be a suitable primary endpoint to predict vaccine effectiveness [19,20].

Among our 92 vaccine recipients, 88 tested positive for anti-S (95.7%), with a median anti-S titer of 52.15 RU/mL (166.88 BAU/mL). Our recorded median titer is quite similar to that of a Jordanian study (170.0 ± 230.0 BAU/mL), but they recorded a lower seroprevalence rate (85.7%) among their Sinopharm recipients [11]. A study from Sri Lanka reported similarly high seroconversion rates (98.8%) by a double Sinopahrm vaccination protocol among similar age groups as in our study, but they measured anti-RBD rather than anti-S levels [21].

Fewer of our participants (67.4%) were positive for nAbs compared to the higher anti-S seropositivity (95.7%). This poor neutralizing ability might imply that other vaccine types might be preferred to Sinopharm if they prove to have higher neutralizing abilities. A German study reported a markedly weak antibody production following a double Sinopharm vaccine compared to mRNA and other DNA-vectored vaccines. However, the same study suggested that owing to the nature of Sinopharm vaccine (whole inactivated SARS-CoV-2 viral particles), the analyses of spike protein reactivity may miss the full extent of immune reactions to this vaccine. Further investigations are required to explore the responses to other viral proteins [22].

Higher seroconversion of nAbs among Sinopharm vaccine recipients was recorded in a study in Sri Lanka, where 81.25% of individuals had ACE2 receptor blocking antibodies, which were similar to the nAbs levels measured in the convalescent sera they tested from COVID-19 patients [21].

The results of a study during the Phase III clinical trial of Sinopharm vaccine declared that the vaccine had a 99% seroconversion rate of nAbs and 100% effectiveness in preventing moderate and severe disease. They also reported that neutralizing virus-specific antibodies were detected in 91.84% of their vaccine recipients on day 28 post second vaccine dose [23].

A study reported that their titers of anti-S (measured by EUROIMMUN QuantiVac ELISA kit as in our study) and titers of neutralizing antibodies (measured by a microneutralization assay) revealed a strong correlation (rs = 0.819), which was very close to the correlation coefficient in our study (0.875, *p* < 0.001) [24]. EUROIMMUN (Germany), the manufacturer of both kits used in our study, stated that the SARS CoV-2 NeutraLISA and the anti-SARS-CoV-2 QuantiVac ELISA (IgG) had an agreement in their qualitative results of 99.1% after excluding borderline results from the calculation [17]. Despite the significant correlation between both antibodies in our study, among the 88 positive anti-S results, 29.5% (n = 26) of anti-S-positive participants were negative for nAbs.

In our study, we generated an ROC analysis to assume cutoff values of anti-S which might predict the presence of nAbs. The ROC curve showed that when the anti-S titer was greater than 40 RU/mL (128 BAU/mL), nAbs were also positive with a sensitivity of 80.6% [95% CI: 68.6%–89.6%], and specificity of 90% [95% CI: 73.5%–97.9%]. This was also supported by our finding in which higher titers of anti-S were found in patients with positive nAbs compared to participants with low anti-S titers who were less likely to have positive nAbs results. This might explain the discrepancy between the seropositivity of anti-S (95.7%) and nAbs (67.4%) levels, where lower anti-spike levels might have only a weak neutralizing ability. This implies that higher anti-S levels might be useful in the prediction of nAbs status of persons (post-infection or post-vaccination) when it is not feasible to test for nAbs, owing to the easier and more available testing methods of anti-S in comparison to the more technically laborious and time-consuming nAbs detection tests. Confirmation of the predictive ability of anti-S requires further research with larger sample size.

Similarly, a Russian study generated a ROC analysis to detect a cutoff for anti-S. In their study, they tested serum samples from participants vaccinated with Sputnik Light vaccine against two viral variants and reported a variant-dependent optimal sensitivity and specificity for their anti-S and nAbs. Their optimal sensitivity for the prediction of nAbs was achieved at anti-S levels of 19.4 and 23.3 BAU/mL for the B.1.1.1 and B.1.617.2 variants, respectively, while their optimal specificity for nAbs detection was achieved at higher anti-S values, that is, 142.7 BAU/mL for both SARS-CoV-2 variants [25]. Our study, however, aimed to detect a cutoff value of anti-S for the prediction of nAbs, regardless of further challenge by viral exposure. We obtained a single value for the cutoff with optimal sensitivity and specificity (40 RU/mL = 128 BAU/mL).

In our study, females had higher titers of anti-S and nAbs than males; however, these differences between genders did not reach statistical significance. Some studies reported comparable anti-S seroprevalence rates between both genders following Sinopharm double vaccination [11,26], while others reported higher seropositivity rates among females [27,28]. In contrast, Markmann et al. reported higher convalescence anti-S and nAb seropositivity among males than females [26]. Differences in disease severity and humoral responses to vaccines have been hypothesized to be influenced by a combination of sex hormone effects on immune cell signaling, X chromosome immune-related gene expression, microRNA levels, and genetic polymorphisms in genes encoding important immunologic proteins such as interleukins [29]. The insignificant results between genders in our study might be attributed to the relatively small sample size of our study. Further research with larger sample sizes should be encouraged.

An overall negative correlation (although insignificant) was seen between age and each of the anti-S titers and IH% of nAbs with insignificant differences in seropositivity rates. Other studies involving elderly participants reported lower seropositivity rates with increasing age [11,22,30]. It is of note, however, that our study did not include children (this vaccine is not yet approved for children) or elderly participants. The median (IQR) age of our participants was 39 (33–45) years, which reflected the nature of our participants, who were all actively working HCWs. It was thus not possible to determine the effect of extremes of age on antibody production following vaccination.

In our present study, no differences were observed in seropositivity rates for either of the antibodies among previously infected COVID-19 patients. This was in contrast to findings of another study, which reported 10.5-fold higher odds of anti-S positivity among previously COVID-19 infected persons [31]. However, we found statistically significant higher titers of anti-S and %IH of nAbs in previously infected COVID-19 participants. Legros et al. reported that both anti-S IgGs and nAbs were detectable at 5–7 days post onset of symptoms in most patients, and they rapidly increased to reach a peak but progressively declined from 40 days post onset [32]. In our study, all participants who reported previous COVID-19 infection also reported that this infection occurred 5 or more months before our sample collection. The long time-lapse might be associated with waning of antibodies, although higher titers among previously infected individuals were also noticed, denoting variable immune response among participants.

According to the WHO, some variants of SARS-CoV-2 with key changes in the spike protein have a reduced susceptibility to nAbs. While nAbs mainly target the spike protein, cellular immunity elicited by natural infection can also target other viral proteins, which tend to be more conserved across variants than the spike protein [10]. The discrepancy between studies regarding the role of past infection might be explained by viral variants at the time of study, variable severity of COVID-19 patients among studies (mild–moderate–severe), and the variation in the time of antibody testing in relation to infection.

In the present study, smoking was not significantly associated with seropositivity rates or serum levels, despite the higher titers of anti-S and %IH of nAbs among non-smokers. Similar results were reported by another Egyptian study on vaccinated HCWs, where anti-S levels were 4.5-fold higher in non-smokers [31]. Kanizsa et al. also reported significantly lower serum S-IgG antibody levels in smoking individuals (31). A recent systematic review analyzed COVID-19 vaccine response in relation to smoking and reported that most studies could not clearly classify whether the reduced antibody levels among smokers was an indication of a reduced immunologic response or due to their more rapid decay [33]. Proposed pathophysiologic mechanisms of reduced immune response among smokers include direct effects on B cells and indirect effects on T cells and antigen-presenting cells, which could affect Ig class switching and/or differential survival of naive B or memory B cells [34].

None of the studied comorbidities, except hypertension, was significantly associated with anti-S or nAbs titers. Hypertensive participants were found to have significantly higher median titers of anti-S (101.18 RU/mL) compared with non-hypertensive ones (42.15 RU/mL), *p* = 0.034. A study from Sri Lanka [21] and another one from Egypt [31] reported a lack of association between comorbidities and anti-S levels among vaccinated participants. Other studies reported lower antibody response to vaccines in persons with hypertension, central obesity, and diabetes, suggesting that altered cardiometabolic features might be involved in the development of immunological response to vaccines [35]. In our study, the small number of hypertensive participants might have affected the significance of our findings, thus more studies with hypertensive vaccinated participants are required to estimate the effect of hypertension on antibody response.

In our study, 35.9% of participants recorded one or more of the following symptoms: fever (n = 6, 6.5%), fatigue (n = 10, 10.9%), headache (n = 12, 13.0%), and pain at the site of injection (n = 16, 17.4%). Adjobimey et al. reported that 93.8% of Sinopharm vaccine recipients had no adverse effects [22]. The results of the safety of the Phase III clinical trial of the Sinopharm vaccine (results from the United Arab Emirates) reported that the vaccine had no serious safety concerns [20]. In our study, post-vaccination headache was significantly higher among those with higher anti-S than those with relatively lower titers (98.82 versus 43.69 RU/mL, *p* = 0.048). Some studies have associated COVID-19 vaccines with post-vaccination hypertension. In a case series of nine patients with stage III hypertension, eight were symptomatic (malaise, headache, tingling in the mouth, and diaphoresis) and had increased blood pressure. All eight patients had received the Pfizer/BioNTech vaccine and the ninth had received the Moderna vaccine. The same study proposed reasons behind this finding, including a stress response (psychological stress due to the public debate on vaccines, in addition to pain response) and hypertension to components of the vaccines such as polyethylenglycol [36]. In another study, six participants, among 113 patients, showed an average rise in systolic or diastolic BP at home by ≥10 mmHg during the first 5 days after receiving the first dose of Pfizer/BioNTech vaccine, compared with the 5 day period immediately preceding vaccination [37]. In our study, the reported headache might thus be due to hypertension following vaccination. However, unfortunately, blood pressure was not measured for our participants in the period following vaccination.

In our study, there was a discrepancy between the patterns of levels of anti-S and nAbs in relation to certain risk factors. For instance, smoking was significantly associated with anti-S, but no similar association was found with nAbs. This indicates that some antibodies might have a binding affinity that is not necessarily neutralizing. An article published in The Lancet explained this finding by the fact that after infection or immunization, there will be antibodies with biological activity, such as neutralization, and others that bind to other regions of the antigen and proteins but whose presence might not correlate with the neutralizing activities. There might also be antibodies that neutralize but are not detected in binding assays; for instance, nAbs directed at regions outside of the receptor-binding domain will not be detected by ELISA tests using the receptor-binding domain as the target antigen. It cannot, therefore, be assumed that the activity in one type of assay, such as neutralization, strictly parallels another, such as binding in an ELISA test [38].

## 5. Conclusions

Around 70.5% of anti-S-positive participants were also positive for nAbs. A significant positive correlation was seen between the titers of both antibodies. Anti-S was found to be a good indicator for the cases with positive nAbs. The ROC showed that when the anti-S titer was greater than 40 RU/mL (128 BAU/mL), nAbs were also positive, with a sensitivity of 80.6% and a specificity of 90%. Hypertensive persons showed a higher immune response, as well as those having post-vaccination headache.

## 6. Limitations

This study was limited by its cross-sectional nature, which did not allow for the study of serial measurements of antibody titers to monitor their temporal decay. The relatively small sample size might have under/overestimated the significance of some risk factors. Further studies with larger sample sizes are recommended. The addition of borderline results, while facilitating statistical interpretation, might have underestimated the actual seropositivity rates. Moreover, the significance of immune protection was not interpreted in relation to the viral sublineages present at that time, owing to the lack of available data.

## Figures and Tables

**Table 1:** Results of SARS-CoV-2 anti-S and nAbs among 92 Sinopharm vaccine recipients.

Variable	Frequency (n = 92)	%
**Qualitative result of SARS-CoV-2 anti-S**		
Positive	88	95.7
Negative	4	4.3
**Titer of SARS-CoV-2 anti-S**		
Median (IQR)	52.15 (31.44–99.12)	
Minimum–Maximum	(2–120)	
**Qualitative result of SARS-CoV-2 nAbs**		
Positive	62	67.4
Negative	30	32.6
**IH% of SARS-CoV-2 neutralizing antibody**		
Median (IQR)	50.62 (24.05–84.36)	
Minimum–Maximum	(9.243–96.34)	

**Table 2:** Testing the association between the SARS-CoV-2 anti-S and nAbs among 92 Sinopharm vaccine recipients.

		SARS-CoV-2 nAbs			
		Negative	Positive	Statistical Test	*p*-Value
		n = 30	n = 62		
		n (%)	n (%)		
**SARS-CoV-2 anti-S**	**Negative (n = 2)** n (%)	4 (100.0)	0 (0.0)	8.642	0.003 *
	**Positive (n = 88)** n (%)	26 (29.5) **	62 (70.5) ***		

* *p*-value was determined using Fisher’s exact test. ** This group had a mean anti-S titer of 18.6 RU/mL. *** This group had a mean anti-S titer of 62.1 RU/mL.

**Table 3:** Spearman correlation matrix determining the relation of the age with SARS-CoV-2 anti-S and IH% of nAbs among 92 Sinopharm vaccine recipients.

		SARS-COV-2 Anti-S	SARS-CoV-2 nAbs	Age (Years)
**Titer of SARS-CoV-2 anti-S**	Correlation Coefficient (r_s_)	1.000	0.875	−0.096
	*p*-value		0.000 *	0.360
**IH%** **SARS-CoV-2 nAbs**	Correlation Coefficient (r_s_)	0.875	1.000	−0.117
	*p*-value	0.000 *		0.265
**Age (years)**	Correlation Coefficient (r_s_)	−0.096	−0.117	1.000
	*p*-value	0.360	0.265	

* *p*-value < 0.01.

**Table 4:** Quantitative analysis of SARS-CoV-2 anti-S titers and nAbs IH% in relation to the characteristics of 92 Sinopharm vaccine recipients.

	SARS-CoV-2 Anti-S (RU/mL)	*p*-Value	SARS-CoV-2 nAbs (%IH)	*p*-Value
**Gender**				
Male (n = 54)	52.15 (7–121)	0.514	48.54 (−5.52–96.03)	0.937
Female (n = 38)	50.46 (2–121)		51.39 (−9.24–96.34)	
**Smoking**				
No (n = 72)	57.08 (2–121)	0.369	52.14 (−9.24–96.34)	0.191
Yes (n = 20)	46.92 (9–121)		41.22 (−1.80–92.43)	
**Previous COVID-19 infection**				
No (n = 76)	41.08 (2–121)	0.043 *	45.94 (−9.24–96.34)	0.048 *
Yes (n = 16)	76.00 (11–121)		73.63 (3.72–96.03)	

* *p*-value was determined using Mann–Whitney test.

**Table 5:** Association between the seroprevalence of SARS-CoV-2 anti-S and nAbs and some characteristics of 92 Sinopharm vaccine recipients.

KERRYPNX	SARS-CoV-2 Anti-S		Statistical Test	*p*-Value	SARS-CoV-2 nAbs		Statistical Test	*p*-Value
	Negative n = 4	Positive n = 88			Negative n = 30	Positive n = 62		
**Age (years)**								
Median (Range)	42 (40–44)	38.5 (23–60)	156.5 ^a^	0.709	40 (26–60)	38 (23–59)	798.0 ^a^	0.271
	n (%)	n (%)			n (%)	n (%)		
**Gender**								
Male (n = 54)	3 (5.6)	51 (94.4)	0.459 ^c^	0.640	19 (35.2)	35 (64.8)	0.395 ^b^	0.530
Female (n = 38)	1 (2.6)	37 (97.4)			11 (28.9)	27 (71.1)		
**Smoking**								
Yes (n = 20)	2 (10.0)	18 (90)	1.963 ^c^	0.205	9 (45.0)	11 (55.0)	1.786 ^b^	0.181
No (n = 72)	2 (2.8)	70 (97.2)			21 (29.2)	51 (70.8)		
**Previous COVID-19 infection**								
No (n = 76)	3 (3.9)	73 (96.1)	0.169 ^c^	1.00	27 (35.5)	49 (64.5)	1.693 ^b^	0.193
Yes (n = 16)	1 (6.3)	15 (93.7)			3 (18.8)	13 (81.3)		

^a^ Mann–Whitney test, ^b^ chi-squared test, ^c^ Fisher’s exact test.

**Table 6:** Quantitative analysis of SARS-CoV-2 anti-S and %IH of nAbs in relation to the medical history of 92 Sinopharm vaccine recipients.

	SARS-CoV-2 Anti-S (RU/mL)	*p*-Value	SARS-CoV-2 nAbs %IH	*p*-Value
**Diabetes**				
- No (n = 88)	52.15 (2–121)	0.464	50.62 (−9.24–96.34)	0.678
- Yes (n = 4)	67.67 (34–121)		58.78 (37.96–8617)	
**Hypertension**				
- No (n = 82)	42.15 (2–121)	0.034 *	46.80 (−5.52–96.34)	0.054
- Yes (n = 10)	101.18 (14–121)		73.48 (−9.24–94.79)	
**Thyroid Diseases**				
- No (n = 88)	57.08 (2–121)	0.327	51.33 (−9.24–96.34)	0.389
- Yes (n = 4)	34.52 (26–72)		37.22 (19.04–62.22)	
**Asthma or any lung condition**				
- No (n = 84)	49.54 (2–121)	0.280	49.22 (−9.24–96.34)	0.443
- Yes (n = 6)	88.19 (25–121)		71.46 (14.15–91.19)	
**Allergy**				
- No (n = 86)	57.08 (2–121)		51.33 (−9.24–96.34)	
- Yes (n = 6)	39.05 (28–121)	0.868	43.48 (3.72–95.53)	0.931

* *p* < 0.05.

**Table 7:** Association between the seroprevalence of SARS-CoV-2 anti-S and nAbs in relation to the medical history of 92 Sinopharm vaccine recipients.

	**SARS-CoV-2 Anti-S**				**SARS-CoV-2 nAbs**			
	**Negative**	**Positive**	**Statistical** **Test**	* *p*-Value	**Negative**	**Positive**	**Statistical** **Test**	* p-Value
	**n = 4**	**n = 88**			**N = 30**	**N = 62**		
	**n(%)**	**n(%)**			**n(%)**	**n(%)**		
**Diabetes**								
- Yes (n = 4)	0 (0.0)	4 (100)			0 (0.0)	4 (100.0)		
- No (n = 88)	4 (4.4)	84 (95.4)	0.190	1.00	30 (34.1)	58 (65.9)	2.023	0.300
**Hypertension**								
- Yes (n = 10)	0 (0.0)	10 (100.0)		1.00	2 (20.0)	8 (80.0)		
- No (n = 82)	4 (4.9)	78 (95.1)	0.510		28 (34.1)	54 (65.9)	0.812	0.489
**Thyroid Diseases**								
- Yes (n = 4)	0 (0.0)	4 (100.0)			2 (50.0)	2 (50.0)		
- No (n = 88)	4 (4.5)	84 (95.5)	0.190	1.00	28 (31.8)	60 (68.2)	0.576	0.594
**Asthma or any lung condition**								
- Yes (n = 6)	0 (0.0)	6 (100.0)	0.292	1.00	1 (16.7)	5 (83.3)		
- No (n = 84)	4 (4.7)	82 (95.3)			29 (33.7)	57 (66.3)	0.742	0.660
**Allergy**								
- Yes (n = 6)	0 (0.0)	6 (100.0)			2 (33.3)	4 (66.7)		
- No (n = 86)	4 (4.7)	82 (95.3)	0.292	1.00	28 (32.6)	58 (67.4)	0.002	1.00

* *p*-value was determined using Fisher’s exact test.

**Table 8:** Quantitative analysis of SARS-CoV-2 anti-S titers and %IH of nAbs and short-term adverse reactions after Sinopharm COVID-19 vaccination.

	SARS-CoV-2 Anti-S (RU/mL)	*p*-Value	SARS-CoV-2 nAbs (%IH)	*p*-Value
**Fever**				
- No (n = 86)	52.15 (2–121)		49.53 (−9.24–96.34)	
- Yes (n = 6)	55.84 (9–121)	0.994	64.24 (−1.80–85.61)	0.987
**Pain or swelling at the injection site**				
- No (n = 76)	54.46 (2–121)	0.804	51.77 (−5.52–96.34)	0.773
- Yes (n = 16)	52.15 (11–121)		45.13 (−9.24–92.43)	
**Fatigue**				
- No (n = 82)	49.54 (7–121)	0.575	49.53 (−9.24–96.34)	0.471
- Yes (n = 10)	69.23 (2–121)		60.54 (−2.79–95.53)	
**Headache**				
- No (n = 80)	43.69 (2–121)	0.048 *	46.80 (−9.24–96.34)	0.085
- Yes (n = 12)	98.82 (9–121)		77.45 (−1.799–94.54)	

* *p*-value < 0.01.

**Table 9:** Testing the association between the seroprevalence of SARS-CoV-2 anti-S and nAbs and short-term adverse reactions after Sinopharm COVID-19 vaccination.

KERRYPNX	SARS-CoV-2 Anti-S				SARS-CoV-2 nAbs			
	Negative	Positive	Statistical Test	*p*-Value	Negative	Positive	Statistical Test	*p*-Value
	n = 4	n = 88			n = 30	n = 62		
	n(%)	n(%)			n(%)	n(%)		
**Fever**								
- Yes (n = 6)	1 (16.7)	5 (83.3)			2 (33.3)	4 (66.7)		
- No (n = 86)	3 (3.5)	83 (96.5)	2.342 ^a^	0.240	28 (32.6)	58 (67.4)	0.002 ^a^	1.00
**Pain or swelling at the injection site**								
- No (n=76)	1 (6.3)	15 (93.8)	0.169 ^a^	1.00	5 (31.3)	11 (68.8)	0.016 ^b^	0.899
- Yes (n=16)	3 (3.9)	73 (96.1)			25 (32.9)	51(67.1)		
**Fatigue**								
- Yes (n = 10)	0 (0.0)	10 (100)	0.510 ^a^	1.00	1 (10)	9 (90)	2.610 ^a^	0.158
- No (n = 82)	4 (4.9)	78 (95.1)			29 (35.4)	53 (64.6)		
**Headache**								
- Yes (n = 12)	1 (8.3)	11 (91.7)	0.527 ^a^	1.00	1 (8.3)	11 (91.7)	3.701 ^a^	0.095
- No (n = 80)	3 (3.8)	77 (96.3)			29 (36.3)	51 (63.7)		

^a^ Fisher’s exact test, ^b^ chi-squared test.

## Data Availability

Raw data available at the author and will be presented at request.
